# Thoracic Aorta Calcium Detection and Quantification Using Convolutional Neural Networks in a Large Cohort of Intermediate-Risk Patients

**DOI:** 10.3390/tomography7040054

**Published:** 2021-10-28

**Authors:** Federico N. Guilenea, Mariano E. Casciaro, Ariel F. Pascaner, Gilles Soulat, Elie Mousseaux, Damian Craiem

**Affiliations:** 1 Instituto de Medicina Traslacional, Trasplante y Bioingeniería (IMeTTyB), Universidad Favaloro-CONICET, Solís 453, Buenos Aires CP 1078, Argentina; mcasciaro@favaloro.edu.ar (M.E.C.); apascaner@favaloro.edu.ar (A.F.P.); dcraiem@favaloro.edu.ar (D.C.); 2 Cardiovascular Imaging Unit, Hôpital Européen Georges Pompidou, INSERM U970, 75015 Paris, France; gilles.soulat@aphp.fr (G.S.); elie.mousseaux@aphp.fr (E.M.)

**Keywords:** convolutional neural network, artery calcium, thoracic aorta calcification

## Abstract

Arterial calcification is an independent predictor of cardiovascular disease (CVD) events whereas thoracic aorta calcium (TAC) detection might anticipate extracoronary outcomes. In this work, we trained six convolutional neural networks (CNNs) to detect aortic calcifications and to automate the TAC score assessment in intermediate CVD risk patients. Cardiac computed tomography images from 1415 patients were analyzed together with their aortic geometry previously assessed. Orthogonal patches centered in each aortic candidate lesion were reconstructed and a dataset with 19,790 images (61% positives) was built. Three single-input 2D CNNs were trained using axial, coronal and sagittal patches together with two multi-input 2.5D CNNs combining the orthogonal patches and identifying their best regional combination (BRC) in terms of lesion location. Aortic calcifications were concentrated in the descending (66%) and aortic arch (26%) portions. The BRC of axial patches to detect ascending or aortic arch lesions and sagittal images for the descending portion had the best performance: 0.954 F1-Score, 98.4% sensitivity, 87% of the subjects correctly classified in their TAC category and an average false positive TAC score per patient of 30. A CNN that combined axial and sagittal patches depending on the candidate aortic location ensured an accurate TAC score prediction.

## 1. Introduction

Arterial calcification is an independent predictor of cardiovascular disease (CVD) events, morbidity and mortality [1]. Calcium deposits can be observed in several vascular beds [2], but coronary artery calcium (CAC) is probably the most studied biomarker of calcium burden. It is generally quantified using the Agatston score [3] which is calculated detecting calcified lesions in non-enhanced computed tomography (CT) axial images, accumulating their size and weighting them by density [4]. Thoracic aorta calcium (TAC), generally detected in the ascending and descending portions of the aorta during coronary examinations, was also associated with CVD events and death [5]. However, only the proximal portion of the ascending aorta and the distal descending aorta segment have been analyzed because they were visible cardiac CT CAC studies. Although the detection of lesions in the aortic arch and the proximal descending aorta were somehow neglected, previous reports have shown that these regions are prone to calcifications [6]. Moreover, the relationship between presence and extent of aortic calcium and the occurrence of stroke [7] or other cardiovascular events or with all-cause mortality has been well demonstrated [6]. Some studies have also shown that the presence and extent of aortic arch calcifications was associated with non-cardiac events [8,9]. Consequently, the development of automatic tools for the assessment of thoracic lesions deserves further attention to simplify the measurement procedure in clinical routine or in studies including large cohort of subjects.

The detection of CAC using the Agatston score method was widely employed in prospective studies [10]. It usually starts with a semi-automated software that detects contiguous voxels with a fixed minimum threshold of 130 Hounsfield units (HU) [4]. Then, an expert manually validates candidate voxels and associate each lesion to the corresponding vessel (i.e., right or left coronary artery). Although candidate voxel detection is a computationally simple procedure, manual validation is often time-consuming, software-dependent and prone to errors and subjective interpretation [11,12]. This method can also be employed to detect calcium along the thoracic aorta, although the lesions are scattered over a larger area. Identifying these lesions on the aortic wall, avoiding those that are in bifurcations or near the vertebrae, while associating them to the ascending, arch and descending aorta segments might be even more challenging. Our group has developed an automated method to isolate the thoracic aorta, estimate its 3D geometry and detect the calcified lesions of intermediate-risk patients using cardiac CT images [6]. Intermediate-risk patients are defined as individuals with predicted 10-years Framingham risk of CVD events between 5–20% that might benefit from further subclinical testing such as vascular calcium assessment to facilitate decisions about preventive interventions [13]. At the end of the automated method, an expert must manually validate each calcified lesion in a time-consuming procedure. This method was recently used to evaluate a large cohort of intermediate-risk patients [8] and can be improved using automatic classification algorithms based on supervised-learning techniques such as Machine Learning and particularly Convolutional Neural Networks (CNNs).

Some authors attempted a fully automated detection of coronary artery calcifications applying CNNs [14,15]. Isgum et al. possibly made the first attempts to automatically quantify calcium in the thoracic aorta using a supervised-learning scheme [16]. In a low-dose non-ECG-triggered modality, other groups used CNNs to locate the heart and to classify coronary calcifications [17,18], even without a prior lesion segmentation [19]. CNNs were also used to measure extracoronary calcium [20], particularly TAC and valvular calcium [18]. Recent studies have analyzed the performance of CNNs to detect, classify and measure coronary and extracoronary calcium across a wide range of CT acquisition types [21]. CNNs were also used to detect and quantify calcium in abdominal [22] and pelvic vasculature [23]. As far as we know, no other authors have developed a supervised-learning scheme to detect and quantify TAC in the entire thoracic aorta using ECG triggered non-enhanced cardiac CT images from a large cohort of patients.

The objective of this work was to design and evaluated different CNNs architectures to detect and quantify thoracic aorta calcifications. The performance of the proposed solutions was validated on a test dataset independent of the training set for quantification of the thoracic aortic calcium score and classification of patients into risk categories. Different combinations of axial, sagittal and coronal images in single- and multi-input networks (2D and 2.5D) were tested. The system was trained and validated on a large cohort of intermediate-risk patients to finally discuss the selection of the most suitable architecture for automatic TAC score estimation and patient risk stratification.

## 2. Materials and Methods

### 2.1. Study Subjects and Image Acquisition

This was a retrospective study that included subjects that were recruited in the Cardiovascular Preventive Medicine unit of the Hôpital Européen Georges Pompidou (Paris, France) over 3 years from 2009 to 2012, as recently reported [8]. Briefly, all consecutive primary prevention patients at intermediate risk of cardiovascular disease that underwent an extended non-enhanced multislice computed tomography (MSCT) scan for vascular calcium assessment in view of cardiovascular risk stratification program were included. Non-enhanced 64-MSCT images were acquired (prospectively ECG-gated) including the heart and the thoracic aorta (TA) from the top of the aortic arch to the level of the diaphragm [8]. Acquisitions were performed at 120 kVp, with tube current adapted to the patient weight (Light-speedVCT; GE Healthcare). All images were reconstructed with a thickness of 2.5 mm and analyzed using custom software designed in our laboratory to detect, label and calculate the size and position of calcifications in the thoracic aorta (TA) [6]. In the current study, we revised the aortic calcium labeling and aortic geometric measurements from an initial group of 1426 subjects to build a supervised-learning system for TAC estimation. Eleven patients were excluded because geometric or calcification files were missing. The reported effective radiation dose of our acquisition protocol was 1.23±0.14 mSv [9]. All measurements were made by the same expert, blinded to clinical parameters.

### 2.2. Detection of Aortic Calcifications

**Figure 1:**
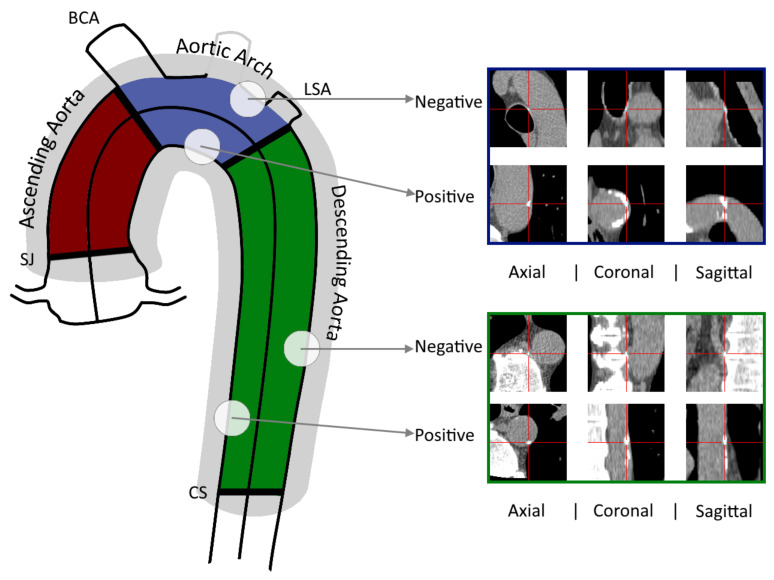
The thoracic aorta was segmented and separated into its ascending, arch and descending portions. Axial, sagittal and coronal images (128 × 128 px) centered at each candidate lesion were reconstructed. Examples of positive and negative calcifications in the regions of the aortic arch and the descending aorta are shown. BCA: brachiocephalic artery, CS: coronary sinus, LSA: left subclavian artery, SJ: sinotubular junction.

Aortic calcifications were analyzed in a final cohort of 1415 subjects. An automated algorithm implemented into a custom software of our laboratory has already detected the thoracic aorta centerline and estimated the aortic diameters at ≈150 centerline points from the sinotubular junction to the descending aorta at the level of the coronary sinus for all subjects. The aortic root and the aortic valves were excluded from this automated detection method. Then, the algorithm identified the position, size and attenuation of every thoracic aorta candidate of calcification. Finally, a manual validation of all the candidates was required from the expert to obtain the corresponding Agatston score [3]. This score was calculated using a categorical weighted value from 1 to 4 depending on the maximum attenuation value registered in each lesion (1: 130–199 HU, 2: 200–299 HU, 3: 300–399 HU and 4: ≥400 HU) multiplied by its area. For each subject, as illustrated in Figure 1, the Agatston TAC score was calculated as the sum of all the lesions’ scores after being also estimated within ascending, arch and descending segment of the thoracic aorta. All thoracic aorta calcification has been manually validated and labeled by an expert using the same platform.

For the present study, we processed again the 1415 scans to add all possible candidate lesions around the aorta and not only those which were marked as positive using our previous automated method. A Python routine (PyCharm [24]) was written to perform this task through the following steps: (i) axial images were binarized with a 130 HU threshold, (ii) an 8-connected region growing algorithm was applied and candidates with an area <1 mm${}^{2}$ were excluded, (iii) the distance between each candidate centroid and the aortic centerline was calculated and those with a distance >1.3 times the aortic radius were also excluded. This distance restriction (gray region in Figure 1) was aimed at preserving the balance of the dataset. These candidates, together with the true aortic labeled calcifications from our previous work, were used to train the CNN.

### 2.3. Datasets and Image processing

Imaging preprocessing and dataset creation were performed using PyCharm. For every patient, lesion candidates were automatically divided into positive aortic calcifications (previously labeled by an expert) and negative aortic calcifications. Negative cases could include coronary, valve or supra-aortic calcifications that were not tagged as aortic lesions and other spots in the trachea or in the vertebrae inside the gray region as shown in Figure 1. Globally, we found 19,790 candidates around the thoracic aorta: 12,041 (61%) were positives and 7749 (39%) negatives.

For each axial slice, three orthogonal patches (coronal, sagittal and axial views) were created around the centroid of each lesion candidate. All images were reconstructed using a bilinear interpolation to achieve a homogeneous and isotropic 0.5 mm spatial resolution. Each candidate image consisted of 128 × 128 pixels (6.4 cm squared side). This size was chosen based on the literature to visualize the entire aortic cross-sectional area and part of the surrounding tissues [18,25]. For statistical purposes, a separate file was also stored for each candidate lesion containing the patient’s ID, the coordinates of the lesion center, the corresponding aortic portion and the Agatston score.

CT scans were randomly divided into two datasets: main set (90% = 70% for training +20% for validation) and test set (10%). A variation of the validation set approach described in [26] was implemented. Accordingly, we randomly shuffled and divided the patients included in the main set to obtain 10 different combinations of training and validation sets. This cross-validation method aimed at informing the variability and confidence intervals of the output metrics in the test set. The test dataset with 10% of the patients remained intact and independent of the training set.

### 2.4. CNN Design

Using Keras based on Tensorflow, six different architectures were evaluated: three in a 2D group and three in a 2.5D multi-input group.

**Figure 2:**
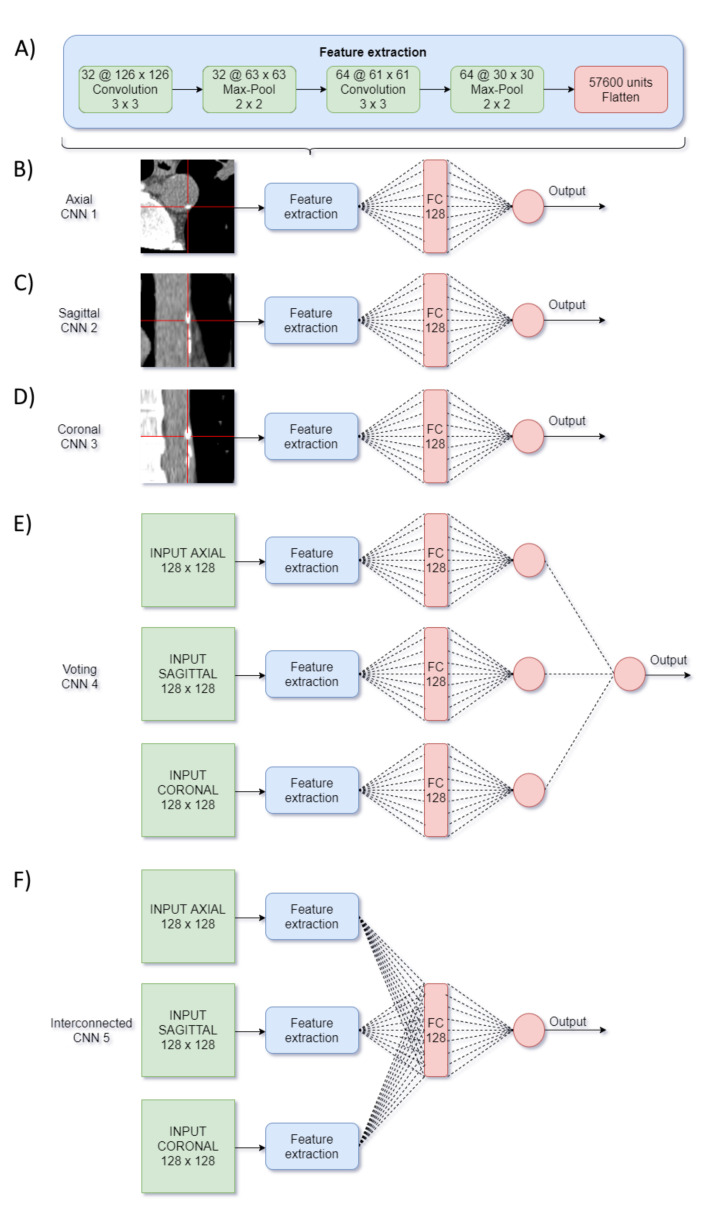
(**A**) Feature extraction block components. (**B**–**F**) CNN 1: Axial network. CNN 2: Sagittal network. CNN 3: Coronal network. CNN 4: Voting network. CNN 5: Interconnected network.

The first 2D group was composed of independent networks numbered 1 to 3, for the axial, sagittal and coronal orthogonal images, respectively. The three networks consisted of 2 convolutional and max-pooling blocks and one fully connected (FC) layer with 128 neurons as shown in Figure 2B–D.

The second group of networks was composed by three multi-input architectures numbered 4 to 6. Networks #4 and #5 were 2.5D multi-input and used the three orthogonal input images simultaneously. They shared the same convolutional and pooling layers from the first 2D group but differed in the FC ones (Figure 2E,F).

**Figure 3:**
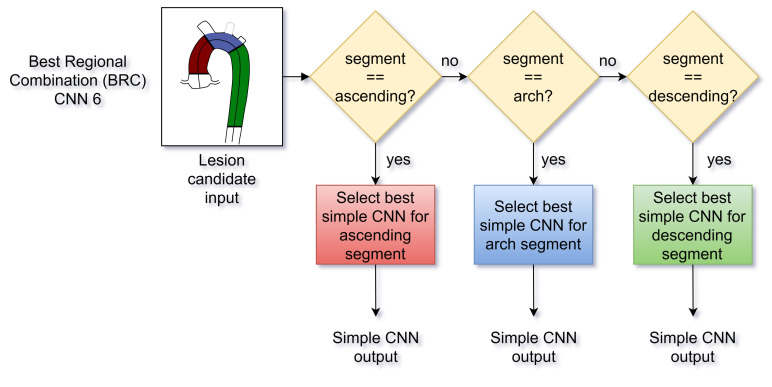
CNN 6: Best Regional Combination architecture.

- Network #4 was based on [17] and was named “voting-network” because the processing of the three images remained independent until the last layer, where a neuron obtained as input a single value from each one, and hence voted among them.
- Network #5 was named "interconnected-network" and was inspired by $CNN_{2}$ in [18]. It combined the information of the three processed images by a concatenation and a FC layer.
- Network #6 was named the Best Regional Combination (BRC) network and was conceived to improve the independent prediction of the single-input CNNs from the first group with a lesion location feature. Accordingly, using the 10 validation sets, the F1-Score of the 3 trained networks number 1-to-3 was compared with respect to the position of the candidate lesions in terms of the aortic region (ascending, arch and descending aorta). Then, the BRC network classified the lesions choosing either the CNN number #1, #2 or #3 depending on the candidate location (Figure 3).

Training consisted of 90 epochs with a mini-batch size of 32. Elastic Net Regularization [27] was included in every layer, using a value of 0.02 in L1 and 0.001 in L2. In FC layers a Dropout of 20% was performed [28]. The activation function used in all layers was ReLU, except in the output neuron where the sigmoid was used, and the cost function was binary cross-entropy. The threshold for the output neuron probability was set at 0.5. Loss, accuracy and F1-Score of the validation set were taken into account during the training process and for model selection.

### 2.5. Statistics and Evaluation Metrics

The main evaluation metric employed to compare the performance of the different architectures was the F1-Score value weighted by TAC in a lesion-by-lesion scheme. This metric took into account the size and the density of the calcifications within the resampling repetitions. First, the F1-Score values of the three 2D CNNs numbered 1 to 3 (axial, sagittal and coronal) were compared within each aortic segment (ascending, arch and descending). Second, F1-Score values of these three architectures and the three 2.5D CNNs numbered 4 to 6 were compared for the whole thoracic aorta. F1-Score boxplots (median and interquartile range) were built for each CNN and were compared using the Kruskal–Wallis non-parametric test followed by a Wilcoxon post hoc test.

Additional information was reported for the resampling repetition corresponding to the median F1-Score value for each of the 6 CNNs, including true and false positives by calcification number, area and TAC score, together with TAC sensitivity. Regarding the patient-by-patient analysis for a clinical evaluation, subjects were separated into four risk groups according to their TAC scores: (i) low risk (TAC ≤ 10), (ii) intermediate risk (10 < TAC ≤ 100), (iii) high risk (100 < TAC ≤ 400) and (iv) very high risk (TAC > 400). The agreement between the actual risk category (manually validated) and each CNN prediction was assessed with the number of patients that were reclassified using Cohen’s linearly kappa [29] and the intraclass correlation coefficient [30]. The association of the reference and BRC predicted TAC values were represented using both a linear regression and a mountain plot [31]. The mountain plot represented the difference between the predicted TAC score by the BRC and the actual value in the abscissa and the patient percentile in the Y axis. A *p*-value below 5% was considered significant. Figures were built with R Studio 1.4 (Boston, MA, USA) and JMP 14 (SAS Institute, Cary, NC, USA) was employed for statistical comparisons.

## 3. Results

Training consisted of approximately 45 h for the 5 networks and the 10 datasets. Relevant clinical information about the 1415 patients included in this study (75% men, 57 ± 9 y.o.) is summarized in Table 1. The cohort accounted 54% of smokers, 47% of hypertensive and 82% of hypercholesterolemic subjects. The average Framingham risk score at 10 years was 9.5%. In terms of aortic calcium, 42% of the subjects remained in the CVD risk group 1 (TAC < 10), whereas ≈20% were homogeneously distributed in the other three groups. In 10% of the patients reserved for the test set (N = 141), no significant differences were observed in risk factors with respect to the training group (N = 1274).

Table 2 shows the number and total area of candidate lesions found in the thoracic aorta of the patients, together with the positive lesions (calcifications) tagged by the expert. Most of both negative candidates and calcifications were found in the descending aorta, followed by the aortic arch and the ascending portion. Total areas followed a similar distribution. Globally, 61% of the candidates corresponded to positive calcifications, being the aortic arch the most balanced portion.

**Figure 4:**
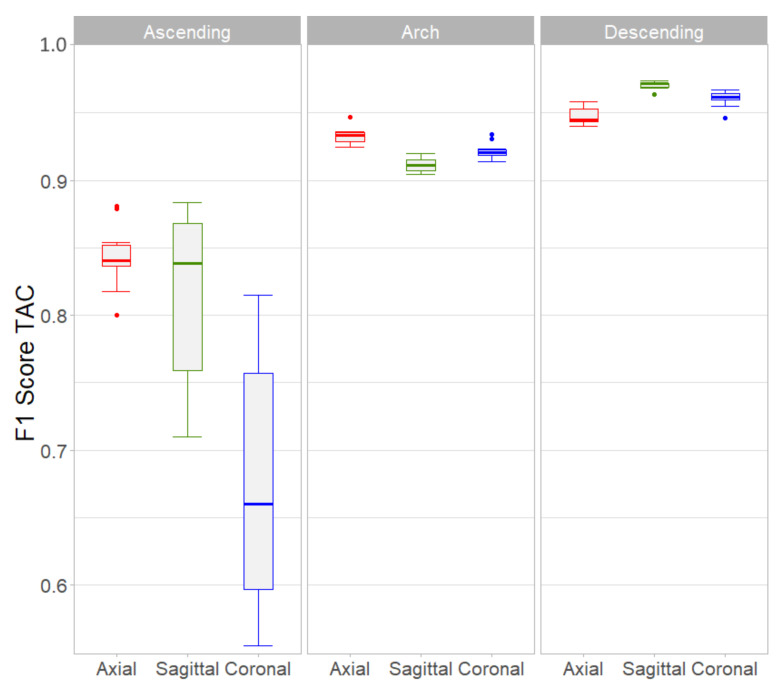
Boxplots of F1-Score weighted by TAC for each of the three single-input architectures on each of the three aortic segments. The Axial architecture was the best in the ascending and arch portions, whereas the Sagittal architecture had a better performance for the descending aorta.

Figure 4 represents the performance of each of the three single-input 2D architectures per aortic segment in terms of the F1-Score weighted by TAC using boxplots to show the variability of the resampling repetitions. In the ascending aorta, Axial and Sagittal CNNs outperformed the Coronal network (*p* < 0.001). Since the interquartile range of the Axial network was one third of the Sagittal one (0.05 vs. 0.14, respectively), CNN number 1 was the best choice for the ascending aorta. The F1-Score values of the Axial CNN were the highest within the aortic arch (*p* < 0.001). In the descending aorta, the Sagittal CNN showed the best results in terms of F1-Score (*p* < 0.001).

**Figure 5:**
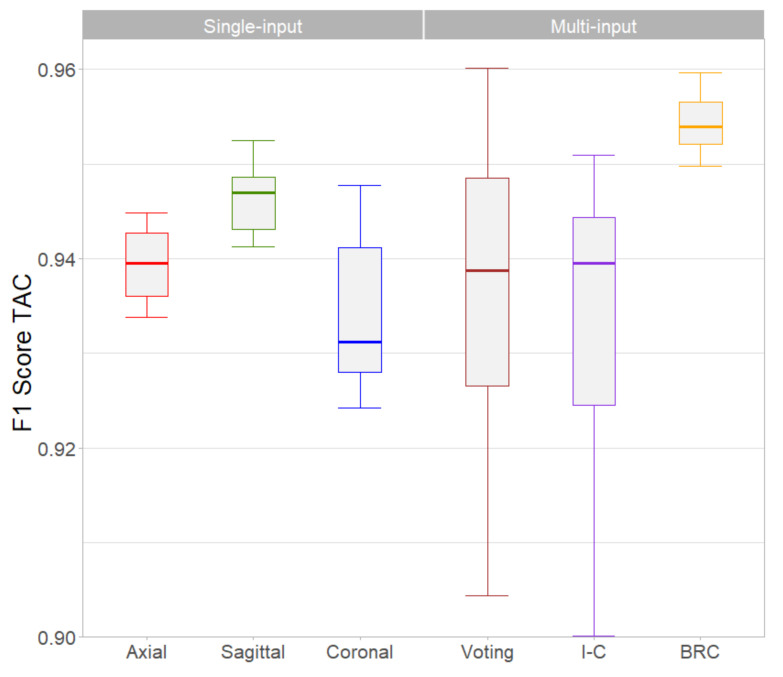
Boxplots of F1-Score weighted by TAC for each architecture. The BRC showed the best performance since its median value was the highest and its variance the lowest. BRC: Best Regional Combination network. I-C: Interconnected network.

The comparison of the single- and multi-input architectures for the whole thoracic aorta is shown in Figure 5. Based on the single-input CNN performance, the best regional combination architecture (BRC) combined the Axial CNN for detection of lesions in the ascending or arch segments and the Sagittal CNN for the descending aorta. The BRC CNN had the highest F1-Score with respect to the other networks (*p* < 0.001). In the multiple individual comparison, the Sagittal CNN outperformed the Axial (*p* < 0.01), Coronal (*p* < 0.01) and Interconnected (*p* < 0.05) networks. The other combinations did not show statistical differences.

On Table 3 other metrics besides the F1-Score were included to compare the different architectures. In general, the BRC network outperformed the rest, although the Axial network was slightly better regarding the false positives. BRC detected 1151 true positives over 1200 positive candidates and a cumulative TAC of 54,516 over 55,425. The false positives corresponded to an average increment of 7.8% TAC score per patient. Its sensitivity was 98.4% and the F1-Score value 0.954. Mean TAC risk kappa values were 0.88 and excellent ICC values of 0.998 were found.

**Figure 6:**
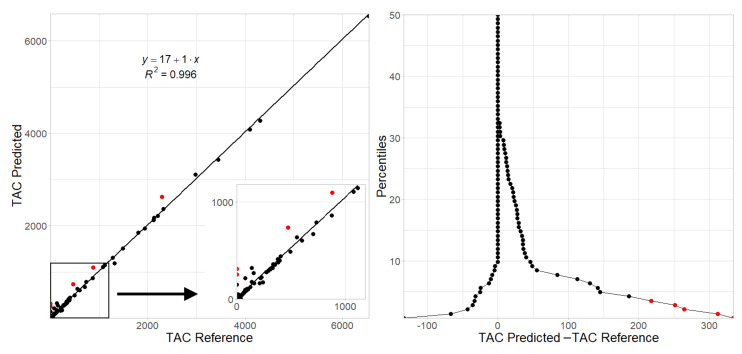
**Left**: Regression plot of reference vs predicted TAC score values for patients ($y=1.0x+17,r^{2}=0.996,n=141$). **Right**: Mountain plot (also called folded empirical cumulative distribution plot) showing differences between predicted and reference TAC score values by patient percentile. Red points indicate patients with TAC score differences >200. TAC predictions were calculated from the BRC network that took into account the position of the lesions within the thoracic aorta segments. BRC: Best Regional Combination network.

Table 4 shows in detail the performance of the BRC network for the 141 patients in the test set. In the reclassification process, 87% (N = 123) of the patients remained in the correct category. It is important to look closely at what happened to the 13% (N = 18) of misclassified patients. Among them, 17 patients were classified above their reference group: ten from group I to II, three from group II to III and only one from group III to IV. Three subjects were misclassified from group I to III because the CNN detected the ligamentum arteriosum as a calcified lesion (TAC reference values were 0 and the CNN predicted 145, 251 and 312). As shown in Figure 6, a good correlation between the reference and estimated TAC was observed. The mountain plot indicates that the TAC prediction tended to overestimate the reference scores values, although for more than 90% of the subjects this TAC score difference was below 50. Among the five patients where this TAC difference was above 200 (red points), two were due to the ligamentum arteriosum misclassification and the other three had a reference TAC > 400 (TAC scores of 469, 876 and 2293).

## 4. Discussion

In this work, different architectures using 2D and 2.5D convolutional neural networks were proposed to detect and quantify thoracic aorta calcifications, evaluating their ability to estimate the Agatston TAC score in a large cohort of intermediate-risk patients. The network that obtained the best performance combined axial images to detect calcifications in the ascending aorta and aortic arch and sagittal images for lesions in the descending aorta. This network, called Best Regional Combination (BRC) had a median F1-Score of 0.954 and a sensitivity of 98.4% on the image per image evaluation. Additionally, it correctly classified 87% of the patients in the test group (123 out of 141) in one of the four reference TAC categories, with an average false positive TAC score per patient of 30. Calcifications along the entire thoracic aorta were predominantly concentrated in the descending portion followed by the aortic arch segment. Instead of using 2.5D multi-input images, the particular characteristics of shape, size, density, location and extension of aortic calcifications in each aortic segment required 2D single-input axial or sagittal networks combined in the proposed BRC architecture.

For the classification system, a dataset of ≈20,000 images was created using three orthogonal patches centered on each aortic lesion candidate. Of the six proposed networks, those numbered 1-to-3 were trained with the individual axial, sagittal and coronal images (single-input) whereas in networks 4-to-6 these images were combined using different strategies (multi-input) with the hypothesis that this additional information would improve the detection performance. Network #2 (Sagittal) was the one that yielded the best results in terms of TAC F1-Score among the single-input ones, with less variability and values always greater than 0.94 in the 10 resampling training process (green boxplot in Figure 5). However, this result was heterogeneous when the other segments of the aorta were evaluated, noting that the ascending aorta was the most challenging segment (Figure 4). In this particular aortic portion, the axial network outperformed the other two, although the variability of the repeated test was high, probably due to the scarce number of lesions (8% of the total). The axial network also outperformed the other two single-input CNNs in the aortic arch, whereas the Sagittal network achieved the best F1-Scores in the descending segment. In this latter region, larger calcifications probably comprised several axial planes and therefore the detection was more accurate using a single sagittal image. This finding might agree with another study that reported a reduction of false positives using a combination of axial and sagittal images to detect abdominal aorta calcifications [22].

The multi-input networks #4 and #5 that used the three orthogonal patches simultaneously, did not achieve a better performance than the sagittal single-input network #2 (Figure 5). This was somewhat unexpected, since the strategies of combining 2.5D images either by the voting or the interconnected networks, were supposed to improve the lesion detection. Although in one of the 10 training repetitions, the Voting network number 5 actually achieved a higher F1-Score than the rest, it was outperformed by the BRC network in terms of median F1-Score value and lower variability. The BRC network was the one that showed the best F1-Score values because it combined the most efficient single-input CNNs after the regional evaluation (Figure 4): the Axial network was used to detect calcifications in the ascending aorta and the aortic arch, while descending aorta lesions were identified using the Sagittal network. Moreover, the BRC network surpassed the rest in terms of sensitivity and true positive TAC values per patient, ranking second behind the axial network in terms of false positives TAC value (Table 4). A similar performance was observed in terms of patient risk classification based on four groups of TAC scores, where the BRC network TAC intraclass correlation score was the highest and its kappa value was almost equal to the Axial network. These results indicate that the problem of detecting aortic calcifications requires specific networks that take into account the heterogeneity in the presence and extent of lesions in the different aortic segments.

Other groups have obtained results comparable to those presented here in terms of TAC detection but in patients undergoing lung cancer screening [16,18] or radiotherapy planning [32]. Our study employed cardiac CT images, the modality considered gold-standard for the detection of calcium using the Agatston method. Our large cohort of intermediate CVD risk patients has also the advantage of including the entire thoracic aorta, with the entire aortic arch included, which is generally beyond limits in conventional CAC studies. This original dataset allowed us to extract valuable information concerning the peculiarities of aortic calcifications that are clearly different from coronary lesions in terms of quantity, distribution, size and shape.

Our strategy to overcome the initial imbalance in our dataset (99.8% of negatives vs 0.2% of positives) was to restrict the candidate detection around the thoracic aorta. Other authors have also applied similar approaches, segmenting the thoracic aorta [33], schemes based on multi-atlas and registration [14,16] or heuristic methods to isolate the heart [15,19]. Other reports proposed a sequential scheme [18,32] where a first CNN is trained with balanced mini-batches to pre-classify valid candidates and a second CNN performs a more refined classification. We are well aware that the next step will be to validate the aortic segmentation by such neural networks, so that eventually all the segmentation and geometric analysis associated with the identification of the different segments and the quantification of aortic calcifications can be done at the same time [34].

Some limitations of our study should be mentioned. First, our retrospective study was conducted in a single center with the same CT equipment. This allowed us to scan the entire thoracic aorta including the aortic arch and to ensure a homogeneous cardiac CT acquisition protocol but we are aware that the incorporation of images from a second center should be carried through. Our cohort consisted of intermediate-risk patients with a high prevalence of TAC (65%) and cannot be extrapolated to a general population. However, this number of calcifications helped us to correctly train the CNNs, particularly in the ascending aorta where lesions are scarce. Finally, only thoracic aorta calcifications above the sinotubular junction were detected in this first study, although coronary, valvular and supra-aortic lesions are visible in our dataset. In particular, valvular calcification is of great importance for intermediate-risk patients, but we decided to start with TAC lesions labeled in previous works of our group. Additional measurements are currently in progress to explore the remaining regions and advance into an extended automatic recognition of cardiovascular calcifications.

To summarize, this work proposed several 2D and 2.5D CNN architectures to detect and quantify TAC in a large cohort of intermediate-risk patients using cardiac CT images. The network that showed the best performance combined axial images to detect calcifications in the ascending aorta and aortic arch and sagittal images in the descending aorta. The imaging dataset was built by taking advantage of a previous work of our group where lesions were manually labeled, and the geometry of the thoracic aorta had been assessed in a large cohort of patients. The next step will be to combine aortic calcium quantification and geometry determination in a single combined network.

## Figures and Tables

**Table 1:** Clinical information for the 1415 patients. More than 58% of the patients presented a TAC score greater than 10.

Clinical Information	Mean, N	(SD), %
Age, y.o.	57	(9)
Male gender	1066	75
Hypertension	661	47
Hypercholesterolemia	1161	82
Current or past smoking	760	54
Framingham Risk Score at 10 years, %	9.5	5.6
CVD Risk I (TAC <= 10)	592	42
CVD Risk II (10 < TAC <= 100)	258	18
CVD Risk III (100 < TAC <= 400)	257	18
CVD Risk IV (TAC > 400)	308	22

**Table 2:** Number and total area of candidate lesions and positive calcifications for the three segments of the thoracic aorta.

Aortic	Candidates		Positives		Positives/
Portion	Number, #	Area, cm${}^{2}$	Number, #	Area, cm${}^{2}$	Candidates
ascending	1508 (8%)	333.8 (13%)	931 (8%)	133.3 (9%)	62%
arch	6489 (33%)	782.3 (29%)	3168 (26%)	488.6 (23%)	49%
descending	11793 (59%)	1553.1 (58%)	7942 (66%)	1025.5 (68%)	67%
Total	19790	2669.2	12041	1647.4	61%

**Table 3:** For each architecture we chose the network that represented the median F1-Score value. In the test set of *n* = 141 patients, 90 subjects (64%) had TAC > 0. We found 1200 lesions (an average of 8.5 lesions per patient) and a cumulative TAC of 55,425 (an average of 393 per patient). Percentages of true and false positives for lesion detection and TAC were calculated with respect to 1200 and 393, respectively. The highest values in each row were highlighted in bold type. AS: Agatston Score.

	Axial	Sagittal	Coronal	Voting	Interconnected	BRC
True positives, *n* (%)	1095	1124	1136	1128	1129	**1151**
	(91.3%)	(93.7%)	(94.7%)	(94.0%)	(94.1%)	**(95.9%)**
False positives, *n* (%)	148	143	182	**140**	158	174
	(12.3%)	(11.9%)	(15.2%)	**(11.7%)**	(13.2%)	(14.5%)
True positive Area	96.8	99.4	100.4	100	100.3	**101.0**
per patient, mm${}^{2}$ (%)	(93.9%)	(96.4%)	(97.5%)	(97.1%)	(97.3%)	**(98.0%)**
False positive Area	**7.94**	9.4	13.4	9.8	10.0	9.1
per patient, mm${}^{2}$ (%)	**(7.7%)**	(9.1%)	(13.0%)	(9.5%)	(9.7%)	(8.8%)
True positive TAC	371	381	385	384	385	**387**
per patient, AS (%)	(94.4%)	(97.0%)	(98.0%)	(97.8%)	(97.9%)	**(98.4%)**
False positive TAC	**26**	32	48	34	34	30
per patient, AS (%)	**(6.7%)**	(8.0%)	(12.1%)	(8.7%)	(8.7%)	(7.8%)
TAC Sensitivity	0.944	0.970	0.979	0.978	0.979	**0.984**
TAC F1-Score	0.938	0.946	0.933	0.947	0.947	**0.954**
TAC Risk Kappa,	**0.879**	0.863	0.846	0.868	0.850	**0.878**
value (95% CI)	**(0.822–0.935)**	(0.802–0.924)	(0.766–0.915)	(0.808–0.928)	(0.786–0.915)	**(0.821–0.936)**
TAC Score ICC,	0.991	0.996	0.983	0.995	0.995	**0.998**
value (95% CI)	(0.987–0.993)	(0.994–0.997)	(0.976–0.988)	(0.993–0.997)	(0.992–0.996)	**(0.997–0.999)**

**Table 4:** Reclassification confusion matrix for the median BRC CNN. Among the 141 patients, the classifier assigned the 87% to the correct category. An overestimation by one CVD risk category occurred in 11% of the patients and only 2% had their risk overestimated by two CVD risk categories. Conversely, a single patient has been underestimated by only by 1 category.

	CNN Prediction					
		I	II	III	IV	Total
Reference	I	42	10	3	0	55
	II	1	34	3	0	38
	III	0	0	21	1	22
	IV	0	0	0	26	26
	Total	43	44	27	27	141

## Data Availability

The data presented in this study are available on request from the corresponding author.
